# An enzyme-activatable dual-readout probe for sensitive β-galactosidase sensing and *Escherichia coli* analysis

**DOI:** 10.3389/fbioe.2022.1052801

**Published:** 2022-10-31

**Authors:** Yifang Huang, Weiwei Feng, Guo-Qiang Zhang, Yuling Qiu, Linlin Li, Liqiu Pan, Nannan Cao

**Affiliations:** ^1^ Department of Clinical Laboratory, The First Affiliated Hospital of Guangxi Medical University, Nanning, China; ^2^ Key Laboratory of Clinical Laboratory Medicine of Guangxi Department of Education, Guangxi Medical University, Nanning, China; ^3^ Department of Gastroenterology, Meizhou People’s Hospital, Meizhou, China; ^4^ Department of Laboratory Medicine and Guangdong Engineering and Technology Research Center for Rapid Diagnostic Biosensors, Nanfang Hospital, Southern Medical University, Guangzhou, China; ^5^ Key Laboratory of Bioactive Materials, State Key Laboratory of Medicinal Chemical Biology, Key Laboratory of Functional Polymer Ministry of Education, and College of Life Sciences, Nankai University, Tianjin, China; ^6^ Guangxi Key Laboratory of Thalassemia Research, Guangxi Medical University, Nanning, China; ^7^ Department of Laboratory Medicine, The Second Affiliated Hospital of Guangzhou University of Chinese Medicine, Guangzhou, China

**Keywords:** dual-readout probe, β-galactosidase sensing, *Escherichia coli* detection, antibiotic resistance analysis, enzyme reaction

## Abstract

Rapid and accurate sensing of β-galactosidase (β-gal) activity is particularly critical for the early detection of many diseases and has become a topic of interest in recent years. However, most traditional probes for β-gal sensing often suffer from the disadvantages of narrow dynamic range, low reaction efficiency and are only employed with either colorimetric or fluorescence sensing. Furthermore, β-galactosidase sensing based assay for efficient detection and antibiotic resistance analysis of *Escherichia coli* (*E.coli*) is not available. Here, an enzyme-induced probe assay was reported for dual sensitive fluorescence and colorimetric measurement of β-gal activity, and was further employed for detection of *Escherichia coli* and their antibiotic resistance analysis. The DCM-βgal probe was virtually non-emissive in aqueous solution, while it could be activated by β-gal to produce bright emission. Under optimized conditions, DCM-βgal displayed high sensitivity, selectivity and rapid response to β-gal with a low detection limit of 1.5 × 10^−3^ U ml^−1^. Importantly, this assay was successfully applied to sensitive detection of *E. coli* cells with a fast detection process within 5 h and a low detection concentration of 1 × 10^3^ CFU ml^−1^. Furthermore, the enzyme-activatable assay was also successfully applied for high throughput *E. coli* antibiotic resistance analysis. The DCM-βgal strategy is applied for the first time on the detection of *E. coli* cells and their antibiotic resistance analysis. It is provided with the advantages of high selectively, a simple operation, low cost and rapid detection. The detection platform can also be extended to analyze the level of β-gal in other types of cells or biological samples. Overall, the simple, effective and dual-readout assay holds promise for efficient sensing of β-gal activity and provides a potential tool for *E. coli* detection and their antibiotic resistance analysis.

## Introduction

Enzymes play important roles in a variety of biological processes and have served as crucial biomarkers for disease diagnosis and monitoring. Rapid sensing of the specific enzymes activity is emerging as a critical strategy for accurate disease diagnostics and developing simple, effective sensing system has gained considerable attention in past decades. Among these specific enzymes, β-galactosidase (β-gal) is widely known as a common kind of hydrolase in cells and the main biological function of β-gal is to remove galactose residues from substrates (Komatsu and Urano, 2015). In recent years, β-gal has attracted extensive attention as a vital enzyme biomarker because it has been shown to be overexpressed in senescent cells, tumor cells as well as in *Escherichia coli* (*E.coli*) (Munoz-Espin and Serrano, 2014; Chen et al., 2016; Gu et al., 2016; Adkins et al., 2017; Nishihara et al., 2018). Therefore, developing efficient methods to detect β-gal with high efficiency and specificity is thus of great importance for the early diagnosis of the specific diseases and the identification of *E.coli* infection.

Although a number of approaches have been proposed for detecting the activity of β-gal, but most of them suffer from various disadvantages. For example, colorimetric methods have been suggested as convenient assays for visual detection of the β-gal concentration (Chen et al., 2015; Chen et al., 2016; Adkins et al., 2017; Jia et al., 2018). However, most of these enzyme-induced colorimetric strategies often do not have broad color changes enough for quantitative measurement of the enzyme activity. Electrochemical methods are sensitive enough to measure β-gal activity (Adkins et al., 2017; Wang et al., 2017). Unfortunately, they mostly rely upon advanced instrumentation and require cumbersome processes. Furthermore, the above methods are not suitable for the assessment of endogenous β-gal activity *in vivo*. In contrast to colorimetric and electrochemical methods, fluorescent sensors have attracted extensive attention because of their simplicity, high sensitivity, high signal-to-noise ratio, and their ability for imaging β-gal *in vivo* (Jiang et al., 2017; Fu et al., 2019; Gu et al., 2019; Zhang et al., 2019). However, most traditional fluorescent probes suffer from the disadvantages of background fluorescence interference, narrow dynamic range and long response time, which can greatly hamper their application in biological analysis. Thus, it is highly desirable to develop a fluorescent probe capable of detecting β-gal with an improved dynamic range and enhanced reaction efficiency.

*E.coli* contamination remain major public health challenge and substantial health burden worldwide, accounting for most urinary tract infections and over 90% of food and waterborne diseases (Oliver et al., 2005; Jones et al., 2008; Scharff, 2012). On the other hand, the increasing trend of antibiotics resistance poses another significant threat to public health. Therefore, the rapid and sensitive detection of *E.coli* concentration and *E.coli* antibiotic resistance has been a crucial research topic and is critical to the early diagnosis and prevention of *E.coli* infection. The current gold-standard method, culturing and plate counting, is accurate and reliable for *E.coli* detection. However, it is quite time-consuming and laborious, requiring at least 2 days from sampling to results and even longer for antibiotic resistance analysis (Rompre et al., 2002). Another widely used and promising method for *E. coli* detection is determination of β-gal activity. β-gal is a well-known intracellular enzyme that is encoded by the lacZ operon in *E. coli* cells and it has been widely used as an indicator for the determination of *E. coli* concentration in drinking water and food samples (Colquhoun et al., 1995; Derda et al., 2013; Burnham et al., 2014; Chen et al., 2015; Chen et al., 2016). A number of strategies and probes have been developed to detect β-gal in *E. coli*. Unfortunately, those strategies often take several hours for reaction and are only employed with either colorimetric or fluorescence sensing. Moreover, the requirement of multiple steps of T7 bacteriophage infection and complex chemical reaction in some assays further restrict their clinical application (Chen et al., 2015; Chen et al., 2016; Wang et al., 2017).

**FIGURE 1:**
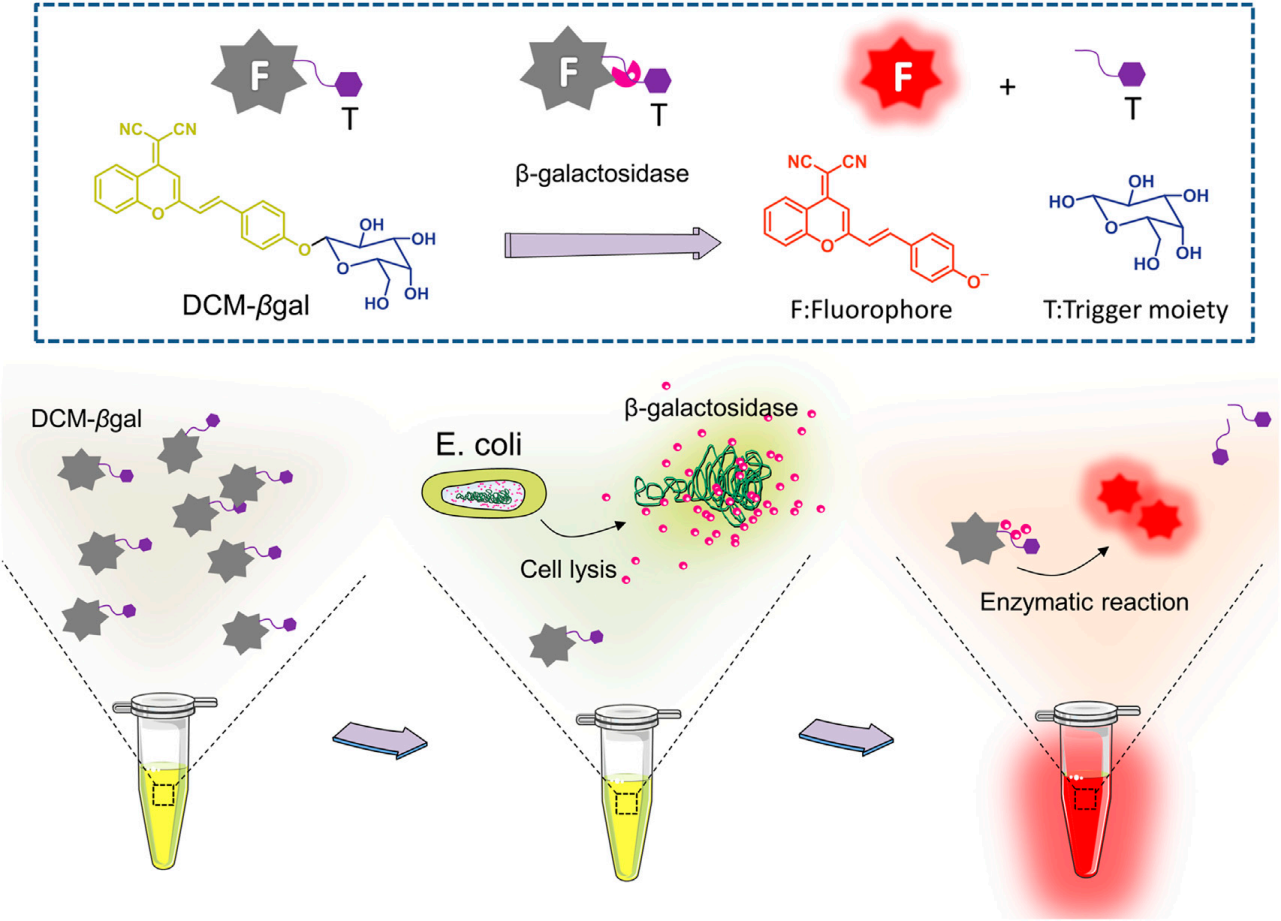
Schematic illustration of enzyme-induced probe assay for measuring of β-gal concentration and detection of *Escherichia coli* (*E.coli*). DCM-βgal probe consists of two parts: DCM-OH as the reporter unit and a β-gal-responsive unit. In the presence of β-gal, the responsive unit is cleaved and the DCM group shows enhance fluorescent emission and an obvious color shift from yellow to red. For *E.coli* detection, the bacterial cells are lysed to release β-gal for enzymatic reaction. The concentration of *E. coli* cells is correlated with the colorimetric shift and the fluorescence intensity of emission spectra peak.

Here, we utilized a simple and effective probe, DCM-βgal, for dual sensitive fluorescence and colorimetric measurement of β-gal activity. For this sensing probe, dicyanomethylene-4H-pyran (DCM) chromophore is utilized as reporter, and a β-gal-responsive group (β-galactopyranoside) as the enzyme-active trigger (Figure 1 **)** (Gu et al., 2016). DCM-βgal emits weak background fluorescence in aqueous buffer. In the presence of β-gal, the β-galactopyranoside unit is cleaved, releasing the DCM group, resulting in a remarkable fluorescent emission in the near-infrared region (NIR) and an obvious color shift from yellow to red. The probe enables sensitive and accurate detecting of β-gal activity with high-selectivity and simplicity. Unlike rhodamine derivates, there is only a single cleavage site in DCM-βgal, which makes the kinetic analysis much easier. The sensing strategy was employed to detect *E. coli* in aqueous solutions using β-gal activity as indicator. The bacterial cells were lysed by lysis solution to release β-gal for enzymatic reaction. Once released, β-gal catalyzed the β-gal cleavable unit in DCM-βgal to generate DCM and induced a colorimetric and fluorescence change **(** Figure 1 **)**. The concentration of *E. coli* cells was directly correlated with the colorimetric shift and the fluorescence intensity of emission spectra peak. DCM-βgal possesses several advantages, including a fast response speed, a high light-up ratio and a good sensitivity towards β-gal, making it a promising strategy for rapid sensing of β-gal.

## Experimental section

### Materials

β-Galactosidase (β-Gal), horseradish peroxidase, alkaline phosphatase, papain, pepsin, and α-mannosidase were purchased from Beyotime (Shanghai, China). Lysozyme, glucose, bovine serum albumin, γ-globulin, transferrin, mannitol tryptophan and proline were purchased from Solarbio (Beijing, China). Ciprofloxacin, ampicillin, kanamycin, dimethyl sulfoxide (DMSO), Polypropylene pyrrolidone K30 (PVP-K30, MW = 40,000) and all other chemicals and solvents were purchased from Sigma-Aldrich (St. Louis, United States). Luria-Bertani broth (LB) medium was purchased from BD Biosciences (San Jose, CA). All aqueous solutions were prepared with Milli-Q water (≥18 MΩ, Milli-Q, Millipore).

### Synthesis of DCM-βgal

DCM-βgal was synthesized according to the processes described in Supplementary Scheme S1 in Supplementary Material. The substrate Ac4β-Gal-Ph-CHO (320 mg, 0.71 mmol, 1 equiv) was dissolved in 15 ml of acetonitrile followed by the addition of piperidine (70 μl, 0.71 mmol, 1 equiv) and dicyanomethyl chromone (DCMC) (221 mg, 1.06 mmol, 1.5 equiv). Reaction mixture was stirred at 70°C for 2 h and monitored by thin layer chromatography (Petroleum ether: Ethyl acetate = 2: 1). Upon completion, the reaction mixture diluted with Ethyl acetate (100 ml) was washed with saturated solution of 0.5 M HCl (100 ml). The organic layer was separated, washed with brine, dried over Na_2_SO_4_ and evaporated under reduced pressure. The orange residue was purified by column chromatography on silica gel (Petroleum ether: Ethyl acetate = 4: 1) affording Ac4β-Gal-DCM as an orange solid. The residue was dissolved in DCM and MeOH (15 ml, v/v = 1:2), and the MeONa (10 mg, 0.35 mmol, 0.5 equiv) was added into the solution. The mixture was stirred for 50 min, at room temperature and monitored by thin layer chromatography (DCM: MeOH = 10: 1). Upon completion, the reaction mixture was evaporated under reduced pressure. The orange residue was purified by column chromatography on silica gel (DCM: MeOH = 10: 1) affording the target product DCM-βgal as an orange solid (272 mg, 41% yield for two steps). And the NMR data is same as the literature reported (Gu et al., 2016).

### UV-vis and fluorescence spectral measurements

DCM-βgal solution was prepared at a concentration of 100 µM in a 2 ml total volume of reaction buffer (pH 7.4, 30% DMSO and 70% PBS). 1 U ml^−1^ β-gal was added into DCM-βgal solution and the mixture was gently agitated and incubated at 37°C for 15 min. UV-vis absorption spectra for DCM-βgal and reaction product were respectively analyzed on a PerkinElmer Lambda 25 UV/vis Spectrometer. Photoluminescence (PL) spectra were analyzed on a PerkinElmer spectrfluorometer LS55 with a wavelength range of 550–800 nm. Fluorescence quantum yields were detected on a Hamamatsu absolute PL quantum yield spectrometer C11347 Quantaurus-QY.

### Analytical procedures for detection of β-gal

The stock solution (100 mM) of DCM-βgal was prepared in DMSO and the work solution (100 μM, fDMSO% = 30 vol%) was diluted from the stock solution. Different concentrations of β-gal solution (10 µl) were added into work solution (1 ml) and incubated at 37°C for 15 min. The fluorescence intensity at 675 nm and colorimetric results were recorded for the enzymatic products using PerkinElmer spectrofluorometer LS55.

### Photobleaching analysis

1 U ml^−1^ β-gal was added into DCM-βgal solution and the mixture was incubated at 37°C for 15 min. Then enzymatic products were treated with high density bright-light exposure (20 W). The fluorescence intensity of the solution was monitored from 0 to 45 min of exposure.

### Bacterial culture

A single colony of *E. coli* was selected from a LB plate and added into LB liquid medium and incubated overnight at 37°C under in the orbital shaker of 200 rpm agitation. The *E. coli* cells was harvested by centrifugation at 6000 g for 3 min and re-suspended in PBS buffer. The centrifugation process was repeated for three times. The re-suspended bacterial solution was then serially diluted into various concentrations for further use. The diluted *E. coli* solution was plated on LB agar plate to confirm the visible counts (CFU ml^−1^).

### Detection of *E. coli* using DCM-βgal based assay

Bacteria suspension with different concentrations was centrifugated by 6000 g for 3 min and discarded the supernatant. Subsequently, 0.7 ml lysis buffer containing 1 mg ml^−1^ lysozyme, 1% (w/v) sodium chloride, 0.2% (w/v) glycerin, 0.01% (w/v) PVP-K30 was added to the bacterial precipitate, mixed gently and incubated at 37°C for 30 min. The dissolved suspension was then added with 100 μM DCM-βgal and 0.3 ml DMSO and incubated at 37°C for 15 min after gently mixed. The fluorescence intensity at 675 nm and colorimetric results were recorded for the enzymatic solution using PerkinElmer spectrofluorometer LS55.

### Two-step detection of *E. coli* using DCM-βgal based assay

Different concentrations of bacterial solutions (100 μL, 1×10^5^, 5×10^4^, 1×10^4^, 5×10^3^, and 1 × 10^3^ CFU ml^−1^) were incubated in LB broth (900 µl) at 37°C for 1–3 h with 200 rpm agitation. PBS buffer without bacteria cells was taken as a negative control. After incubation, bacteria suspension was centrifugated and the bacterial precipitate was lysed by 0.7 ml lysis buffer as above. The dissolved suspension was added with 100 μM DCM-βgal and 0.3 ml DMSO and incubated at 37°C for 15 min. The fluorescence intensity and colorimetric results of solutions were recorded.

### Bacteria antibiotic resistance analysis

For bacteria antibiotic resistance sensing, LB broth medium was added with different antibiotic drugs (ampicillin, kanamycin, or ciprofloxacin) with varying concentrations (0, 10, 20, 30, 40, and 50 μg ml^−1^). Then, *E.coli* solutions (10^5^ CFU ml^−1^) were added in LB mixtures at 37°C for 3 h with 200 rpm agitation in a constant temperature shaker. After incubation, bacteria suspension was centrifugated and the bacterial precipitate was lysed by 0.7 ml lysis buffer as above. The dissolved suspension was then added with 100 μM DCM-βgal and 0.3 ml DMSO and incubated at 37°C for 15 min after gently mixed. Then, the supernatant was transferred to 96-well plate and the fluorescence intensity of solutions was analyzed by Cytation 5.

### Statistical analysis

All continuous data was presented as means ± standard deviation. Data was statistically analyzed using t-test. *p* < 0.05 was considered statistically significant.

## Results and discussion

### Spectroscopic properties and optical response to β-gal

**FIGURE 2:**
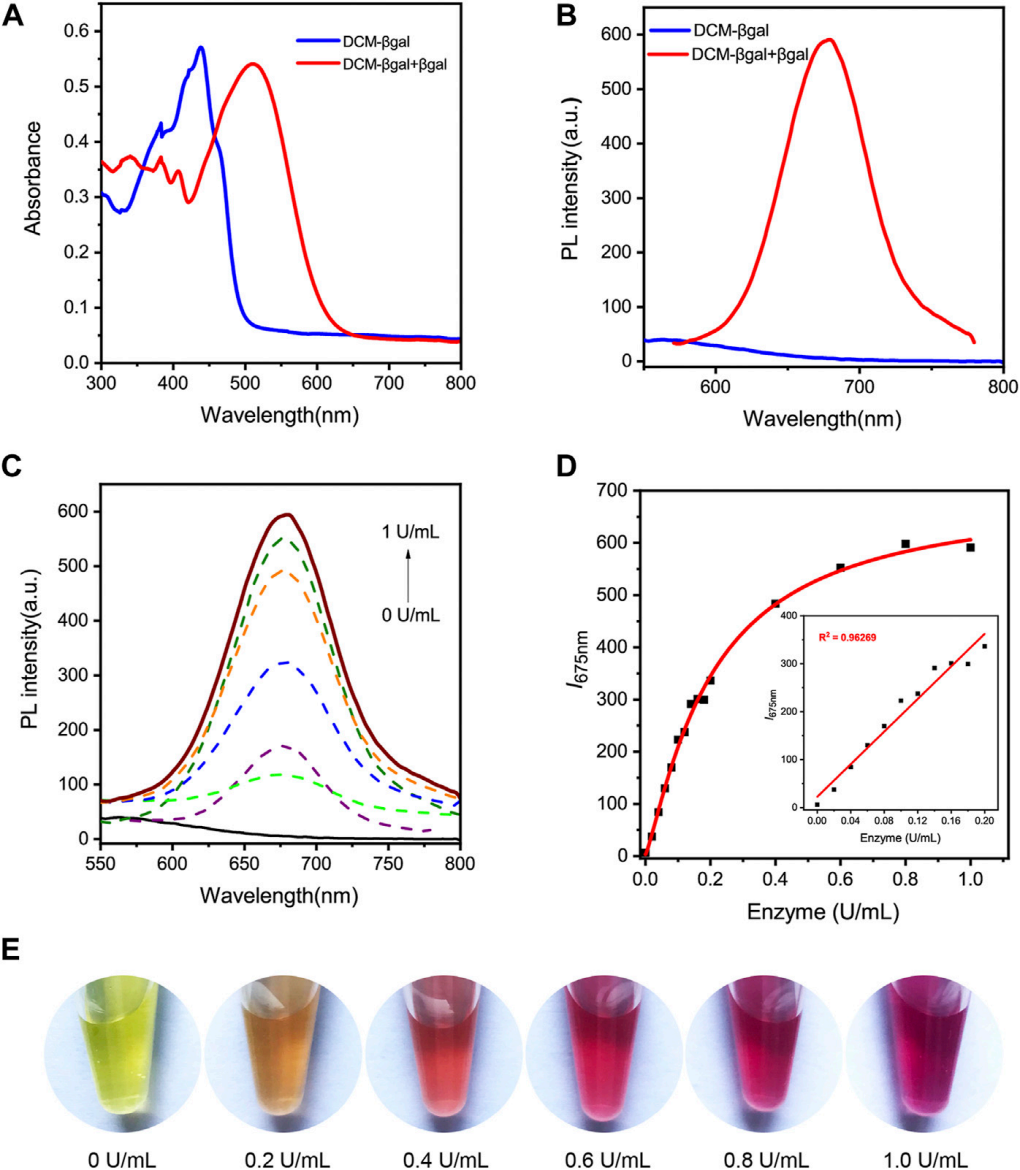
Spectral properties of DCM-βgal. **(A)** Normalized UV-vis absorption of DCM-βgal (100 μM) and DCM-βgal (100 μM) incubation with β-gal (1 U ml^−1^) in aqueous solution (PBS/DMSO = 7:3, v:v, pH = 7.4, 37°C). **(B)** Photoluminescence (PL) spectra of DCM-βgal (100 μM) and DCM-βgal (100 μM) incubation with β-gal (1 U ml^−1^) in aqueous solution (PBS/DMSO = 7:3, v:v, pH = 7.4, 37°C). **(C)** Emission spectra of DCM-βgal upon addition of different concentrations β-gal (0–1 U ml^−1^), λex = 535 nm. **(D)** The peak intensity of DCM-βgal at 675 nm toward various concentrations of β-gal (0–1 U ml^−1^). Inset: the linear relationship between I_675 nm_ and β-gal concentration in a range from 0 U ml^−1^ to 0.2 U ml^−1^ **(E)** The photographs of DCM-βgal upon addition of various concentrations of β-gal.

The basic chemical structure and the principle of DCM-βgal probe for β-gal activity sensing are shown in Figure 1. The synthetic route of DCM-βgal is display in Supplementary Scheme S1 in Electronic Supplementary Material. DCM derivatives are well-known as laser dyes that produce intense emission in the NIR region and DCM-OH is a commonly used signal reporter because of its special properties: it is a Donor–π–acceptor (D–π–A) molecule and it has a hydroxyl group that can be easily modified by other groups (Gu et al., 2016; Gu et al., 2019). Thus, DCM-βgal probe consists of two parts: DCM-OH as colorimetric and fluorescent reporter and a β-galactopyranoside unit as β-gal-responsive moiety. Firstly, to test the validity of DCM-βgal, its spectral properties were investigated by UV-vis-NIR spectroscopy and photoluminescence (PL) spectrum. As shown in Figure 2A, the absorption spectrum showed that DCM-βgal exhibited an obvious absorption peak at 440 nm. Upon addition of 1 U ml^−1^ β-gal, the absorption peak significantly decreased at 440 nm and a new absorption peak appeared at 535 nm. This new absorption was in accordance with that of DCM-O-, suggesting that DCM-βgal was cleaved by β-gal and resulting DCM-OH generation (Gu et al., 2016). Then, the emission profile of DCM-βgal was detected. Upon excitation at 535 nm, the fluorescence signal of DCM-βgal was very weak while the probe solution showed a remarkable NIR fluorescence at 675 nm in the presence of β-gal (Figure 2B). In particular, an obvious color shifting from yellow to red could be observed, allowing for the qualitative or semi-quantitative detection of β-gal by direct observation and rapid colorimetric analysis.

### Optimization of assay conditions

The reaction solution, temperature and pH are important factors for the sensing system. To obtain the best sensing performance, the optical properties of DCM-βgal were examined in different analytical parameters. Upon addition of 1 U ml^−1^ β-gal, the fluorescence intensity of DCM-βgal at 675 nm was tested in PBS/dimethyl sulfoxide (DMSO) mixtures with different DMSO volume fractions (fDMSO%). As shown in Supplementary Figure S1A,B (Electronic Supplementary Material), the fluorescence intensity of DCM-βgal solution increased quickly with fDMSO% and reached a maximum at fDMSO% = 30 vol%. Specifically, as the DMSO content continued to increase, the fluorescence intensity decreased sharply and DCM-βgal solution showed very weak fluorescence at fDMSO% = 50–100 vol%. Correspondingly, a similar colorimetric change could be observed with the change of fDMSO% (Supplementary Figure S1C in Supplementary Material). Since DCM-βgal is poor solvent in PBS, the addition of DMSO will increase its solubility and induces an increase in fluorescence emission. Thus, PBS solution with 30% DMSO was applied as the optimal reaction solution.

The effect of reaction temperature and pH was then evaluated. The fluorescence response of DCM-βgal to 1 U ml^−1^ β-gal was examined by incubation of the solution from 4°C to 56°C for 15 min. As expected, the fluorescence signal increased with temperature and reached the maximum at 37°C, and then decreased after 37°C (Supplementary Figure S2A in Supplementary Material). Hence, 37°C was selected as optimal reaction temperature. pH is another important factor affecting the rate of enzymatic reaction for the sensing probe. pH was adjusted to the desired value by using 0.1 M NaOH and 0.1 M HCl solutions. The pH was measured with a pH meter. The effect of pH (4.0–11.0) for the emission profiles of DCM-βgal was subsequently evaluated after 15 min of reaction. It can be observed that the fluorescence signal was very weak at pH lower than 6.12 and higher than 8.46, and the maximum fluorescence signal was achieved at pH = 7.59 (Supplementary Figure S2B in Supplementary Material). The result suggested that the enzymatic hydrolysis reaction for DCM-βgal could only be proceeding at the optimal pH range and pH = 7.59 was used as the optimal pH condition. In particular, the color of the solution varied from yellow to red corresponding to temperature and pH of the solution. The distinct color changes of the probe were therefore easily to identify visually (Supplementary Figures S2C,D in Supplementary Material).

Then, the fluorescence signal versus time was analyzed to investigate the enzyme response rate. As shown in Supplementary Figure S3A,B (Supplementary Material), as the incubation with β-gal, the fluorescence intensity of DCM-βgal gradually increased in the early stage and reached the maximum at around 15 min. Moreover, the fluorescence response of β-gal in different concentrations of DCM-βgal was investigated to determine the optimal probe concentration. Increasing concentrations of DCM-βgal (0–140 μM) were incubated with 1 U ml^−1^ β-gal at 37°C for 15 min. As depicted in Supplementary Figure S3C (Supplementary Material), the fluorescent signal increased gradually with increasing the concentration of DCM-βgal and reached a plateau after a concentration of 100 μM. Therefore, a DCM-βgal concentration of 100 μM was selected for further experiments to provide an optimal probe concentration. DCM-βgal displayed similar colorimetric response with the increasing of DCM-βgal concentration and incubation time, suggestive of its feasibility in dual-channel sensing for β-gal (Supplementary Figure S3D,E in Supplementary Material).

### Analytical performance of DCM-βgal to β-gal

We next sought to investigate the analytical performance of DCM-βgal for β-gal detection. The fluorescence response of the probe to varying concentrations of β-gal was examined under optimized conditions. As expected, the fluorescence intensity of solution increased with the increasing of β-gal concentration and a 94.84-fold fluorescence enhancement was observed when incubated with 1 U ml^−1^ β-gal (Figures 2C,E). Notably, a good linear relationship between the fluorescence intensity at 675 nm and β-gal concentration in a range from 0 U ml^−1^ to 0.2 U ml^−1^ was observed (Figure 2D). The limit of detection (LOD) was calculated to be as low as 1.5 × 10^–3^ U ml^−1^ based on the signal of blank tests and the standard deviation, indicating the high sensitivity of the probe for the fast and quantitative detection of β-gal. Photo-stability is another important factor to evaluate the performance of the probe in long-term tracking and bioimaging of enzyme activity (Liu et al., 2016). The photo-stability of DCM-βgal was then assessed by time-dependent photobleaching measurements. 1 U ml^−1^ β-gal was added into DCM-βgal solution and the mixture was incubated at 37°C for 15 min. Then enzymatic products were treated with high density bright-light exposure (20 W). The results showed that rounded to 82% of DCM-βgal fluorescence intensity still remained after bright-light exposure for 30 min, indicating the high photo-stability of DCM fluorophore (Supplementary Figure S4 in Supplementary Material). These features make DCM-βgal a promising candidate for long-term tracking and imaging of β-gal in practical applications.

### The selectivity of DCM-βgal

**FIGURE 3:**
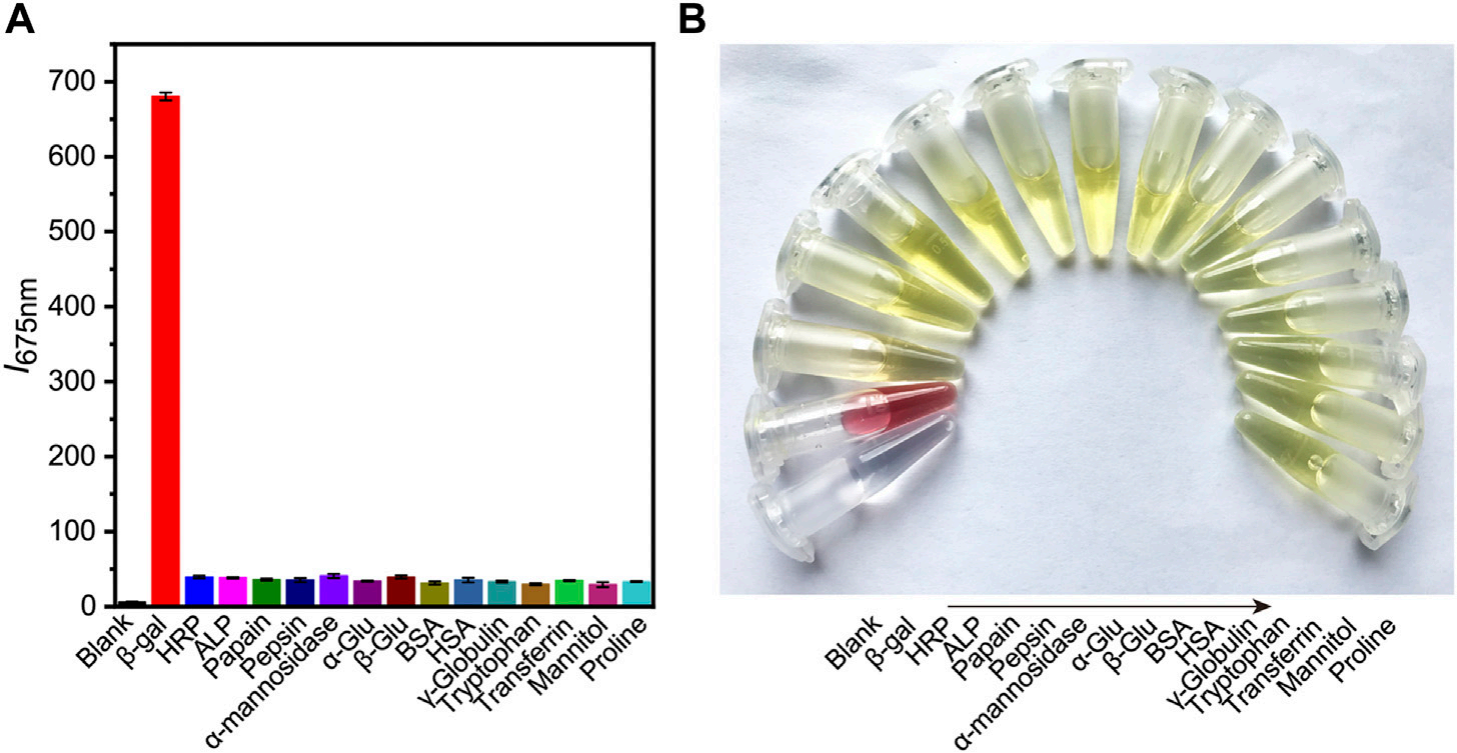
The selectivity of DCM-βgal towards of various biological species. Different types of biological samples are added into work solution of DCM-βgal and incubated at 37°C for 15 min. The fluorescence intensity at 675 nm and colorimetric results of solution are then recorded. **(A)** The fluorescence responses (I_675 nm_) of DCM-βgal to various analytes. Error bars represent the standard deviation of three replicates. **(B)** Corresponding color changes of DCM-βgal treated with different analytes.

Subsequently, the selectivity of probe to β-gal sensing was then evaluated. Control experiments were conducted to investigate the selectivity of DCM-βgal towards of various biological species, including common enzymes (horseradish peroxidase, alkaline phosphatase, papain, pepsin, and α-mannosidase), bioactive molecules (glucose, bovine serum albumin, γ-globulin, transferrin, and mannitol) and amino acids (tryptophan and proline). As expected, an obvious fluorescence enhancement accompanying with an obvious color change from yellow to red was observed in the presence of β-gal **(** Figure 3 **)**. However, negligible fluorescence and color change was observed for other interferents. These results demonstrated the high selectivity of DCM-βgal for β-gal over other competitive analytes, suggesting its promising use as a bioprobe for detecting β-gal in biological systems.

### Application for detection of *Escherichia coli*

**FIGURE 4:**
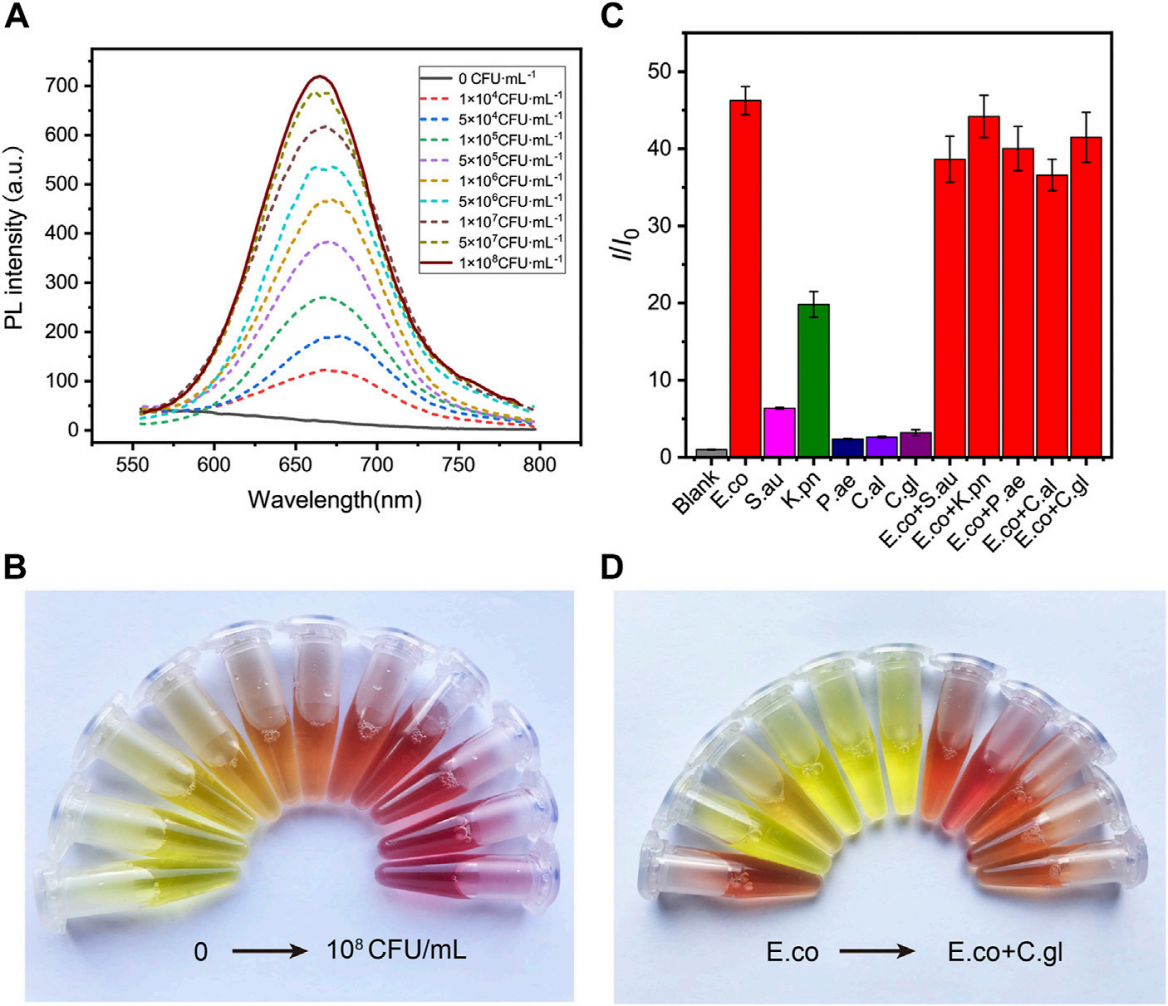
The sensitivity and specificity of DCM-βgal based assay to *E. coli.* **(A)** DCM-βgal based assay for the detection of *E.coli* in the concentration range of 0–1.0 × 10^8^ CFU ml^−1^ and **(B)** its corresponding color changes. Error bars indicate ± SD of triplicate measurements. **(C)** Fluorescence ratio (I/I_0_) of specificity of the DCM-βgal probe against *E.coli*, *S. aureus*, *K. pneumonia*, *P. aeruginosa*, *C.albicans*, *C.glabrata*, *E.coli* + *S. aureus*, *E.coli* + *K. pneumonia*, *E.coli* + *P. aeruginosa*, *E.coli* + *C.albicans* and *E.coli* + *C.glabrata* at concentration of 1.0 × 10^6^ CFU ml^−1^. **(D)** Its corresponding photographs.

Encouraged by the desirable results of DCM-βgal, we next sought to investigate the potential utility of DCM-βgal for detecting β-gal from *E. coli* cells. β-gal is an important bacteria enclosed enzyme encoded by the lacZ gene in *E. coli* cells, and can be used as an indicator for determination of the concentration of *E. coli* (Laczka et al., 2010; Derda et al., 2013; Burnham et al., 2014; Chen et al., 2016). *E. coli* ATCC 25926 was used as a model for investigating the performance of DCM-βgal in *E. coli* detection. Varying concentrations of bacterial cells were firstly lysed, resulting in the release of intracellular β-gal from cells into solution. Then, the solution containing the released β-gal was incubated with DCM-βgal to determine its concentration under optimized conditions. As a result, the *E. coli* concentration could be assessed using the fluorescence intensity of DCM, or directly by the naked eyes. As shown in Figure 4A, the fluorescence intensity and the red color of the enzymatic solution increased with the increasing of bacterial concentration, and distinct signal changes could be reproducibly detected or be distinguished visually for 1 × 10^5^ CFU ml^−1^ of *E. coli* cells (Figure 4B). Compared with previously methods, the DCM-βgal based assay showed a low detection limit and provided a convenient colorimetric readout for visual detection, making it a sensitive and simple strategy for *E. coli* detection.

To test the specificity of the assay to *E. coli*, the responses of the assay to common bacteria strains, including *Staphylococcus aureus* (S.*aureus*), *Klebsiella pneumoniae* (*K. pneumoniae*), *Pseudomonas aeruginosa* (*P. aeruginosa*), *Candida* albicans (*C.albicans*) and *Candida* glabrata (*C.glabrata*), as well as mixtures of different bacteria strains, were investigated. A concentration of 1 × 10^6^ CFU ml^−1^ was used for solutions of each strain or their mixtures. Figures 4C,D shows the fluorescence and colorimetric detection results. No obvious fluorescence signal and color change were observed in S.*aureus*, *P. aeruginosa*, *C.albicans* and *C.glabrata* solutions. In contrast, a significant fluorescence response and distinct color change were detected in *E. coli* solution or in mixtures containing *E. coli*. In particular, the fluorescence intensity of *K. pneumonia* solution was slightly increased and the color of the solution changed from yellow to orange, correspondingly. This result was in accordance with the fact that a certain level of β-gal isoenzymes could be encoded by *K. pneumonia* (Wang et al., 2014). These results demonstrated that the dual-channel assay had a good selectively to β-gal for *E. coli* detection.

**FIGURE 5:**
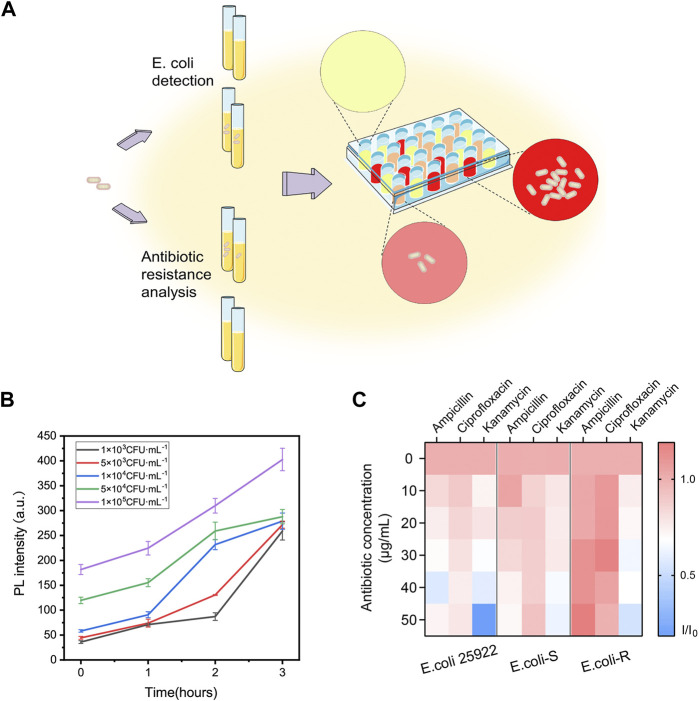
Two-step procedure for *E. coli* detection and high-throughput antibiotic screening using DCM-βgal based assay. **(A)** Schematic illustration of two-step procedure and high-throughput antibiotic analysis. *E. coli* cells are lysed to release intracellular β-gal and the signal generation based on enzyme-response is correlated with the concentration of *E. coli* cells. For antibiotic resistance analysis, *E. coli* cells can be treated with various antibiotic concentrations and fluorescence ratio of reaction products in 96-well plate can be high throughput monitored by Cytation. **(B)** Two-step detection of *E. coli* at low concentrations using DCM-βgal based assay. PL intensity (675 nm) with detection time (0–3 h) toward *E. coli* concentration of 1 × 10^3^, 5 × 10^3^, 1 × 10^4^, 5 × 10^4^, and 1 × 10^5^ CFU ml^−1^. Error bars indicate ± SD of triplicate measurements. **(C)** Heatmap plot of fluorescence ratio (I/I_0_) of three types of *E. coli* cells (*E. coli* ATCC 25926, antibiotic-sensitive (*E. coli*-S) and resistant *E. coli* (*E. coli*-R) collected from clinical samples) toward various antibiotic concentrations.

The concentration of *E. coli* cells is important for the enzymatic response, whereas *E. coli* in clinical urine samples is well below 1 × 10^4^ CFU ml^−1^. To improve the clinical performance of the assay, two-step process was employed to detect *E. coli* cells at low concentrations. Pre-enrichment step was utilized to allow for bacterial growth, and the detection step was then used for determination of *E. coli* cells. Low concentrations of bacteria were incubated in LB liquid medium for 1, 2, and 3 h, respectively. The fluorescence intensity and the color change of solution were investigated after bacteria incubation and enzymatic reaction. As shown in Figures 5A,B, a significant fluorescence and obvious colorimetric response could be detected after 3 h pre-enrichment in all concentrations. *E. coli* cells at the concentration of 1 × 10^5^ CFU ml^−1^, 5 × 10^4^ CFU ml^−1^ and 1 × 10^4^ CFU ml^−1^ could also be clearly detected after a pre-enrichment step of 2 h incubation. Compared with previous methods, the assay was a more efficient strategy with the whole detection time less than 5 h (Chen et al., 2017; Wang et al., 2017). As shown in Table 1, several similar assays for *E. coli* detection were summarized and compared. Sensitive methods often require advanced instrumentation and cumbersome processes, limiting their use in resource-limited settings. In contrast, the DCM-βgal assay would be a promising method for rapid detection of *E. coli* for their practicality, simplicity and low-cost. These results indicated that the DCM-βgal assay had a good performance for *E. coli* detection at low concentrations incorporating a pre-enrichment step.

**TABLE 1:** Summary and comparison of techniques for detection of *E. coli* stains.

Method (publication year)	Output	Detection limit	Detection time	Antibiotic resistance analysis	Instruments	Cost	Ref
Our DCM-βgal based assay	Luminescent and Colorimetry	10^3^ CFU ml^−1^	5 h	Yes	Simple	Low	
p-Benzoquinone (2019)	Colorimetry and Electrochemistry	10^4^ CFU ml^−1^	1 h	Yes	Complex	Middle	Sun et al. (2019)
Sequential Immunomagnetic Separation and Paper-Based Isotachophoresis (2019)	Colorimetry	920 CFU ml^−1^	3 h	No	Middle	Middle	Schaumburg et al. (2019)
Electrochemical methods using engineered bacteriophages (2017)	Electrochemistry	10^2^ CFU ml^−1^	7 h	No	Complex	Middle	Wang et al. (2017)
Printed Paper- and Transparency-Based Analytic Devices (2017)	Colorimetry and Electrochemistry	2.3 × 10^2^ CFU ml^−1^	4 h	No	Complex	Middle	Adkins et al. (2017)
Engineered T7lacZ phage (2017)	Colorimetry	10^2^ CFU ml^−1^	7 h	Yes	Middle	Middle	Chen et al. (2017)
Enzyme-induced silver metallization on the surface of AuNRs (2016)	Colorimetry	10^4^ CFU ml^−1^	2 h	No	Simple	Low	Chen et al. (2016)
T7Bacteriophage-conjugated Magnetic Probe (2015)	Colorimetry	10^4^ CFU ml^−1^	2.5 h	No	Middle	Middle	Chen et al. (2015)
On-site phage-mediated detection (2014)	Luminescent	40 CFU ml^−1^	8 h	No	Complex	Middle	Burnham et al. (2014)
Filter-Based Assay (2013)	Colorimetry	50 CFU ml^−1^	Less 4 h	No	Complex	Middle	Derda et al. (2013)
Interdigitated microelectrode arrays (2010)	Electrochemistry	6 × 10^5^ CFU ml^−1^	2 h	No	Complex	Middle	Laczka et al. (2010)
Interdigitated microelectrode arrays (2010)	Electrochemistry	10 CFU ml^−1^	7 h	No	Complex	Middle	Laczka et al. (2010)
Bacteriophage-amplified bioluminescent sensing (2008)	Luminescent	1 CFU ml^−1^	12.5 h	No	Complex	Middle	Ripp et al. (2008)
Bead-based immunoassay (2005)	Electrochemistry	2 × 10^6^ CFU ml^−1^	Less 1 h	No	Complex	Middle	Boyaci et al. (2005)
Bead-based immunoassay (2005)	Electrochemistry	20 CFU ml^−1^	6–7 h	No	Complex	Middle	Boyaci et al. (2005)

### Application for *Escherichia coli* antibiotic resistance determination

The prevalence of bacteria’s antibiotic resistance is a growing global health concern and it is in urgent need to develop a rapid, efficient strategy for the determination of *E. coli* antibiotic resistance (Walsh, 2000). Conventional test for bacteria antibiotic resistance detection requires cumbersome processes and often takes days to weeks for results. Inspired by the high sensing property of the probe, the ability of DCM-βgal based assay for rapidly detection of *E. coli* antibiotic resistance was then studied (Figure 5A). *E. coli* ATCC 25926, antibiotic-sensitive (E. coli-S) and resistant *E. coli* (E. coli-R) collected from clinical samples were used as bacterial model, and the response of the assay to the bacterial was examined in the presence of three common antibiotics, ampicillin, kanamycin, and ciprofloxacin. *E. coli* cells were incubated in LB liquid medium containing different concentrations of antibiotics at 37°C for 3 h. Bacterial cells were lysed and DCM-βgal probe was then added for enzymatic reaction to determine bacteria concentration. In the presence of effective antibiotic drugs, *E. coli* cells exhibited inhibited growth and a greatly reduced level of β-gal. However, *E. coli* cells showed exponential growth in the presence of antibiotic resistance. Varying levels of β-gal resulted in different levels of enzymatic response, thus providing a simple strategy for antibiotic resistance analysis. With the help of a high throughput fluorescence detection system, Cytation, the fluorescence information of 96-well plate could be easily quantified and monitored. As shown in Figure 5C, for *E. coli* ATCC 25926 and E. coli-S, the fluorescence intensity decreased with the increasing antibiotic concentration when incubated with ampicillin, kanamycin, and ciprofloxacin, indicating that these antibiotics were effective drugs for inhibiting cell growth of these strains. However, for E. coli-R, no decreasing trend for fluorescence intensity could be observed with the increasing concentration of ampicillin and ciprofloxacin, demonstrating that these antibiotics could not effectively inhibited cell growth of this strain. The resistance results were in consistent with those of Kirby-Bauer tests, a common conventional test. Thus, this enzyme-activatable assay has the potential to provide a simple and accessible tool for *E. coli* antibiotic resistance analysis.

## Conclusion

In conclusion, we have proposed an enzyme-responsive NIR probe, DCM-βgal, for sensitive measuring and monitoring β-gal activity. DCM-βgal was almost non-emissive in the absence of β-gal, while it could be induced to produce bright emission upon the addition of β-gal. Particularly, the probe also provided a rapid and convenient colorimetric readout for visual assaying of the β-gal activity. The probe displayed high sensitivity, selectivity and rapid response to β-gal. Simultaneously, the probe also had a good photo-stability, and exhibited a good linear relationship with β-gal concentration from 0 to 0.2 U ml^−1^, with a low detection limit of 1.5 × 10^−3^ U ml^−1^. Importantly, this assay was successfully applied to sensitive detection of *E. coli* cells, as well as rapidly determination of the antibiotic resistance profile of *E. coli via* levels of the color response. The strategy provided several advantages for *E. coli* detection, including a fast detection process within 5 h, a low detection concentration of 1 × 10^3^ CFU ml^−1^, a good selectively to *E. coli* sensing and dual fluorescent and colorimetric readout. Further developments include integrating this assay into point of care testing devices, detecting more real and complex samples would provide improved detection outcomes. Overall, the simple, low-cost and friendly-to-user approach provides an accessible tool for efficient quantification of β-gal activity and is promising for *E. coli* detection and antibiotic resistance analysis.

## Data Availability

The original contributions presented in the study are included in the article/Supplementary Material, further inquiries can be directed to the corresponding authors.
