# Supplementary material for: An enzyme-activatable dual-readout probe for sensitive β-galactosidase sensing and Escherichia coli analysis
Source: Front Bioeng Biotechnol. 2022 Oct 31;10:1052801. doi: 10.3389/fbioe.2022.1052801 (PMC9659582; doi:10.3389/fbioe.2022.1052801)
Supplement: Supplementary file 1 [file DataSheet1.docx]

Supplementary Material

# Supplementary Figures


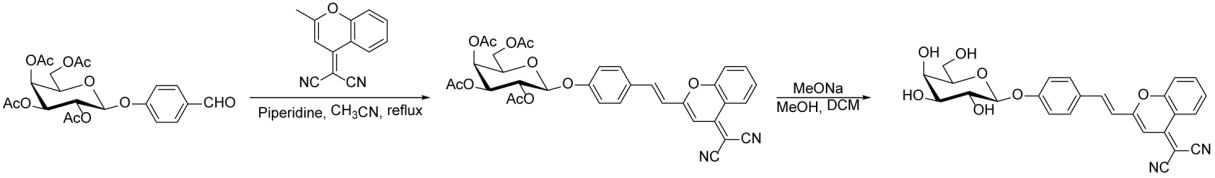


**Scheme S1.** Synthetic route of DCM-βgal probe.


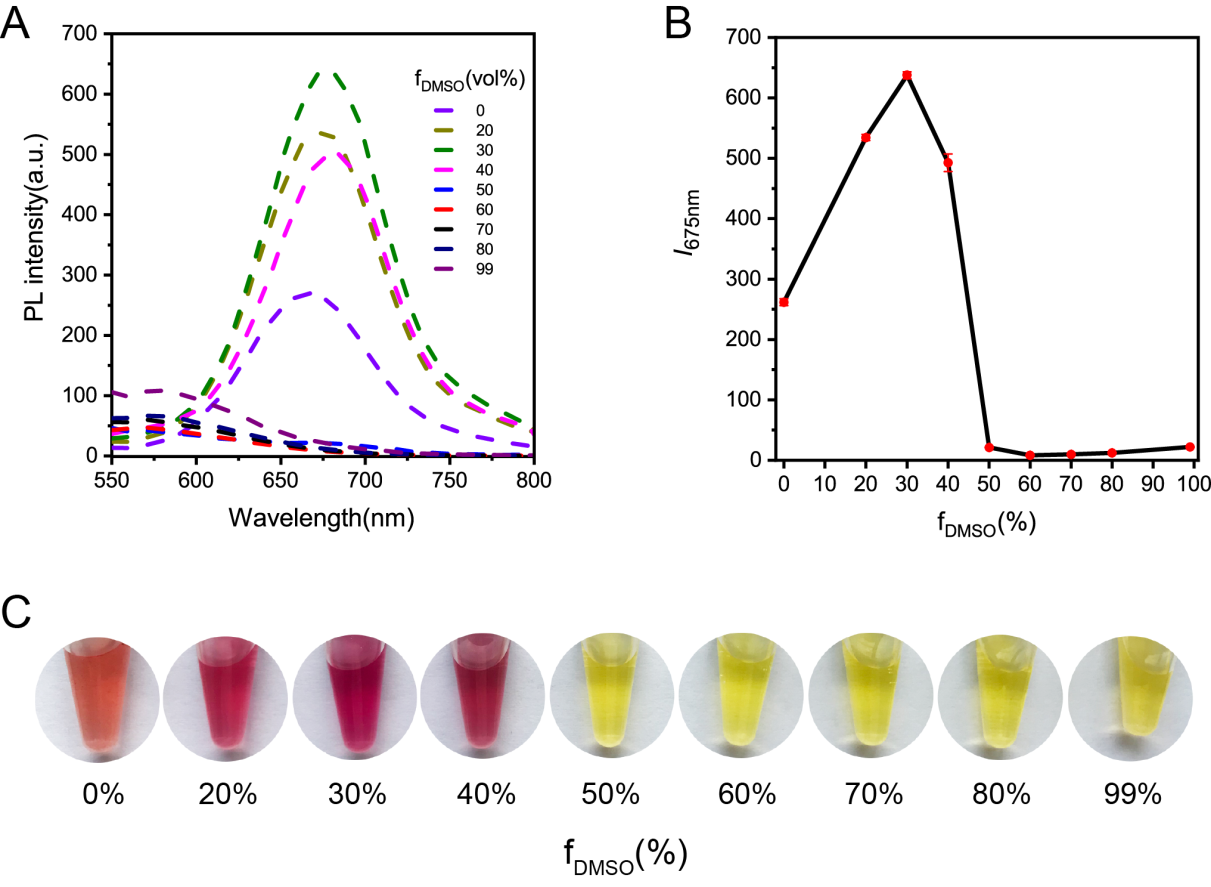


**Supplementary Figure 1.** Optimization of assay reaction in solution with different DMSO volume fractions (fDMSO%). (A) The PL spectra of DCM-βgal (100 μM) incubation with β-gal (1 U•mL^-1^) in DMSO/water mixtures with different DMSO fractions (fDMSO) at 37 ° for 30 min. (B) Plot of peak PL intensity (I_675_ nm) of DCM-βgal (100 μM) incubation with β-gal (1 U•mL^-1^) with various DMSO fractions. (C) Its corresponding photographs.


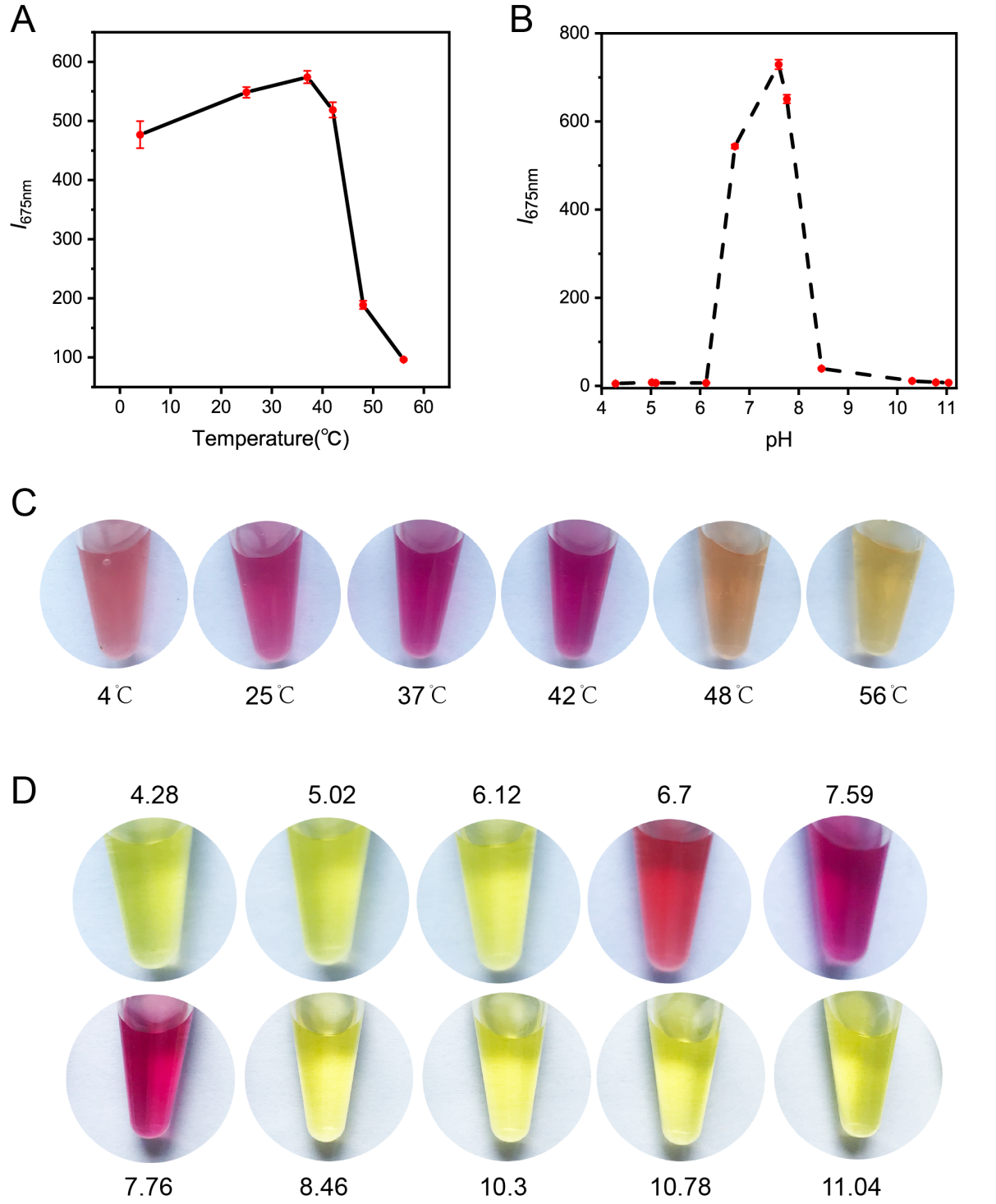


**Supplementary Figure 2.** Optimization of assay reaction in solution with different temperature or pH. (A) Peak PL intensity (I_675_ nm) of DCM-βgal (100 μM) incubation with β-gal (1 U•mL^-1^) at different temperature (4℃, 25℃, 37℃, 42℃, 48℃, 56℃) in aqueous solution (PBS/DMSO = 7:3, v:v) and (C) its corresponding photographs. (B) Peak PL intensity (I_675_ nm) of DCM-βgal (100 μM) incubation with β-gal (1 U•mL^-1^) at different pH (4.28, 5.02, 6.12, 6.7, 7.59, 7.76, 8.46, 10.3, 10.78, 11.04) in aqueous solution (PBS/DMSO = 7:3, v:v, 37℃) and (D) its corresponding photographs.


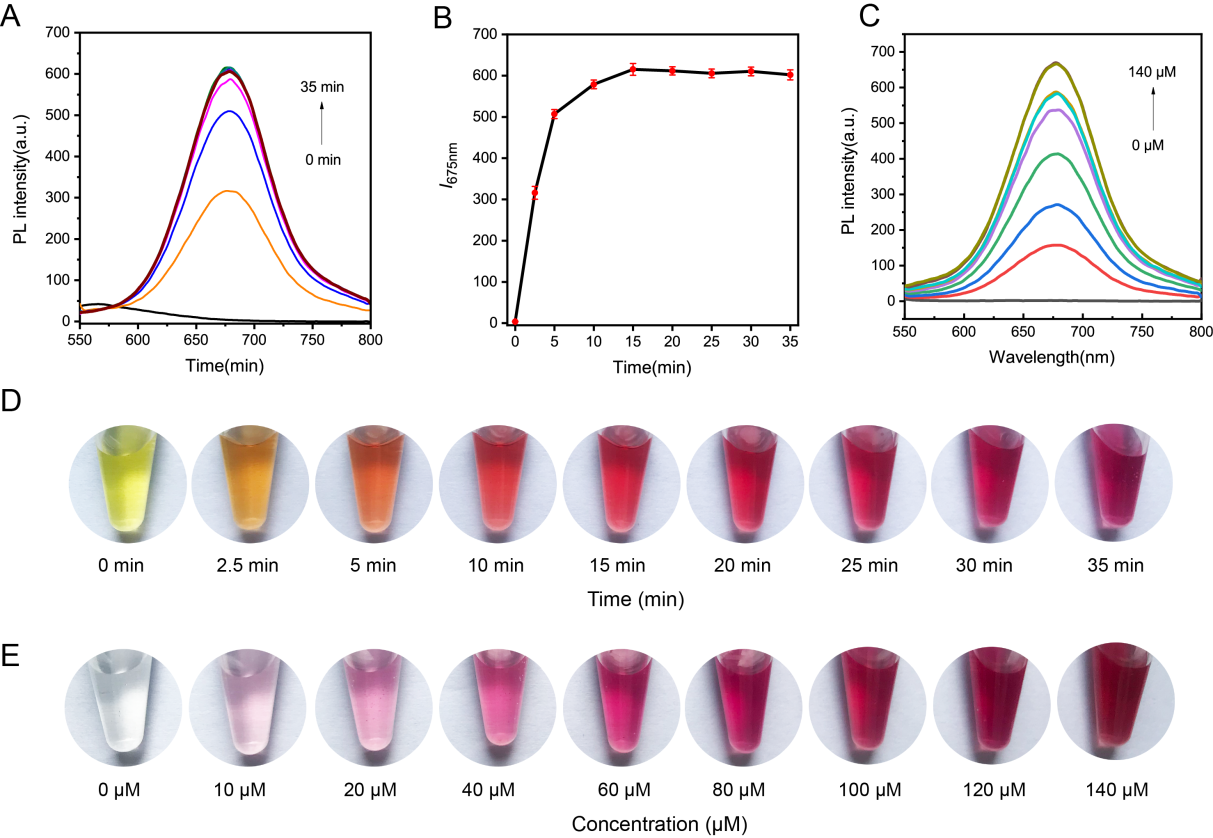


**Supplementary Figure 3.** Optimization of the assay with different reaction time or DCM-βgal concentration. (A) The PL spectra and (B) peak fluorescence intensity (I_675_ nm) of DCM-βgal (100 μM) incubation with β-gal (1 U•mL^-1^) in DMSO/water mixtures as a function of time and (D) its corresponding photographs. (C) Dependence of the PL spectra on varying concentration of DCM-βgal (0, 10, 20, 40, 60, 80, 100, 120 and 140 μM) incubation with β-gal (1 U•mL^-1^) in DMSO/water mixtures for 30min at 37℃ and (E) its corresponding photographs.





**Supplementary Figure 4.** Time-dependent photobleaching measurements for substrates of DCM-βgal incubation with β-gal. The relative ﬂuorescence (I/I_0_) of substrates is monitored under high density bright-light exposure (20 W).
